# Focused electromagnetic high-energetic extracorporeal shockwave (ESWT) reduces pain levels in the nodular state of Dupuytren’s disease—a randomized controlled trial (DupuyShock)

**DOI:** 10.1007/s10103-021-03254-9

**Published:** 2021-01-23

**Authors:** Karsten Knobloch, Marie Hellweg, Heiko Sorg, Tomas Nedelka

**Affiliations:** 1 Plastic, Hand and Reconstructive Surgery, Hannover Medical School, Hannover, Germany; 2 SportPraxis Prof. Dr. Karsten Knobloch, Heiligerstr. 3, 30159 Hannover, Germany; 3 Plastic, Hand and Reconstructive Surgery, Westfalen Klinik, Dortmund, Germany; 4 Department of Neurology, 2nd Faculty of Medicine and Motol Faculty Hospital, Charles University in Prague, Prague, Czech Republic

**Keywords:** ESWT, Shockwave, Therapy, Dupuytren, Nodule, Pain

## Abstract

Dupuytren’s disease is a progressive fibroproliferative disorder of the hand. In the nodular stage of Dupuytren’s disease, pain might limit daily hand activities and progress to finger contractures. Focused electromagnetic high-energetic extracorporeal shockwave therapy (ESWT) may reduce pain in Dupuytren’s nodules (Tubiana N). In this prospective, randomized, blinded, placebo-controlled single center trial, we enrolled 52 patients (mean age, 58.2 ± 9.2) with painful nodular Dupuytren disease Tubiana N. Randomization was done to either (group A) 3 treatments with focused electromagnetic high-energetic ESWT (2000 shots, 3 Hz, 0.35 mmJ/mm^2^/hand, Storz Duolith SD1, *n* = 27) or (group B) placebo ESWT (2000 shots, 3 Hz, 0.01 mJ/mm^2^/hand, *n* = 25) in a weekly interval. Primary outcome was the level of pain on a visual analogue scale (VAS 0–10) at 3/6/12/18 months, secondary outcomes were patient-related outcome measures (DASH score, MHQ score, URAM scale), grip strength, patient’s satisfaction, and Dupuytren’s disease progression over 18 months follow-up. Focused ESWT significantly improved outcomes. Pain was reduced from 3.6 ± 1.8 to 1.9 ± 1.2 at three, to 1.4 ± 0.7 at six, to 1.7 ± 1.6 after 12 months and 1.9 ± 0.8 after 18 months in the intervention group (47% reduction, *p* < 0.05). In the placebo group, pain on VAS increased from 2.2 ± 1.4 to 3.4 ± 1.7 at three, to 3.4 ± 1.8 at six, to 3.4 ± 1.4 at 12 and 3.1 ± 1.1 at 18 months (35% increase, *p* < 0.05). Quality-of-life score tended to improve in the intervention group (MHQ, 77 ± 19 to 83 ± 12; DASH, 12 ± 18 to 10 ± 9) while it deteriorated in the placebo group as Dupuytren’s disease was progressing (MHQ, 79 ± 15 to 73 ± 17; DASH, 6 ± 10 to 14 ± 13). The strength of the affected hand and fingers did not change significantly in either of the groups. Patients’ satisfaction was higher in the intervention group for symptom improvement (56% vs. 12%) and reduction of disease progression (59% vs. 24%). Any Dupuytren-related intervention was performed in 26% in the intervention group and in 36% in the placebo group within 18 months of follow-up (n.s.). Focused electromagnetic high-energetic ESWT can significantly reduce pain in painful nodules in Dupuytren’s disease in an 18-month perspective. (ClinicalTrials.gov Identifier: NCT01184586).

## Introduction

Dupuytren’s disease is a progressive fibromatosis of the palm and flexor side of the hand with similar, but more rare manifestations located on the dorsal PIP joints as knuckle pads (aka Garrod’s nodules) [1] [2], at the foot sole as Ledderhose’s disease [3], or at the dorsum of the penis in Peyronie’s disease. As is customary, Dupuytren starts with nodular manifestation in the palm of the hand and might progress to a pretendinous cord with consecutive finger joint contracture.

When dealing with Dupuytren’s disease, one should differentiate the nodular initial stage from established cords with contractures, since treatment recommendations differ substantially between these two entities [4]. While on cords with finger joint contractures >20° surgical limited fasciectomy [5], percutaneous needle fasciotomy (PNF) [6] or enzymatic collagenase injections [7] are offered country-wise differentially [8] [9] [10], and have been studied quite extensively, in the nodular stage treatment recommendations are scarce. Alternative treatments in nodular stage of Dupuytren’s disease include low-dose radiation therapy, anti-inflammatory, and/or anti-mitotic drugs-like tamoxifen [11].

From a histological point of view, Dupuytren nodules contain whorls of collagen bundles and are densely packed with contractile fibroblasts and myofibroblasts [12]. These highly contractile cells are linked to the fascia matrix through transmembrane integrin receptors. The cytoplasmic tail domains of the alpha beta integrin receptors provide a structural link between extracellular matrix and the actomyosin cytoskeleton. As far as pathogenesis is concerned, abnormal activation of the Wnt signaling pathway as well as microvascular angiopathy with ischemia have been linked to an activation of transforming growth factor ß1 with proliferation of myofibroblasts [13]. TGF-ß is a master regulator of fibrosis [14] acting on multiple cell types driving fibrosis in renal fibrosis as well as in pulmonary fibrosis.

Carla Stecco described [15] a significant higher number of free nerve endings in pathological palmar aponeurosis vs. control suggesting that the palmar aponeurosis is central to proprioception of the hand and that nervous structures are implicated in the amplified fibrosis. Pain can be evident both, in the nodular as well as in the cord stage of Dupuytren’s disease and might deteriorate quality of life substantially.

Extracorporeal shockwave therapy (ESWT) as a noninvasive therapy is using acoustic waves characterized by a sharp, abrupt, and rapid change in pressure as a wave front with a velocity higher than the speed of sound followed by a longer negative tail to elicit a body response. Since the first clinical report on successful kidney stone resolution by high-energetic focused electrohydraulic ESWT on Dec 13, 1980 in The Lancet by Chaussy [16], a substantial number of publications have been done over the past 4 decades on various tissues in regard to the beneficial effects of ESWT. As far as fibrotic tissue is concerned, we hypothesized in 2011 that focused ESWT [17] might be an option, since in plantar Ledderhose’s disease of the foot sole—which resembles the nodular stage of Dupuytren’s of the hand both clinically and histologically—we have demonstrated that Ledderhose’s nodule pain can be significantly reduced by high-energetic, electromagnetic-generated focused ESWT [18]. In line, in penile fibromatosis—aka Peyronie’s disease—a number of randomized-controlled trials reported pain reduction following penile ESWT [19] [20] [21]. Clinically, in Dupuytren’s disease, only two recent case series have been reported for either high-energetic focused ESWT in four patients [22] or radial ESWT in a single case [23] with no long-term follow-up, however underpinning potential beneficial effects of focused and radial ESWT on Dupuytren’s disease on evidence level of IV.

### Hypothesis of this RCT

Based on these aforementioned observations, we hypothesized that focused high-energetic ESWT can reduce pain on painful Dupuytren nodules. To answer this scientific question most adequately, we chose a prospective randomized-controlled study design (Dupuyshock RCT ClinicalTrials.gov NCT01184586).

## Methods

The study protocol was composed according to the most recent CONSORT recommendations for transparent reporting of randomized-controlled trials.

### Ethics and trial design

The study was approved by the IRB at Hannover Medical School. The RCT is directed according to the ethical principles of Good Clinical Practices. It is registered at ClinicalTrials.gov with the identifier NCT01184586 (https://clinicaltrials.gov/ct2/show/NCT01184586).

### Study design

DUPUYSHOCK is a prospective, single center, randomized, blinded, placebo-controlled clinical trial with 1:1 parallel group randomization.

### Participants

**Fig. 1:**
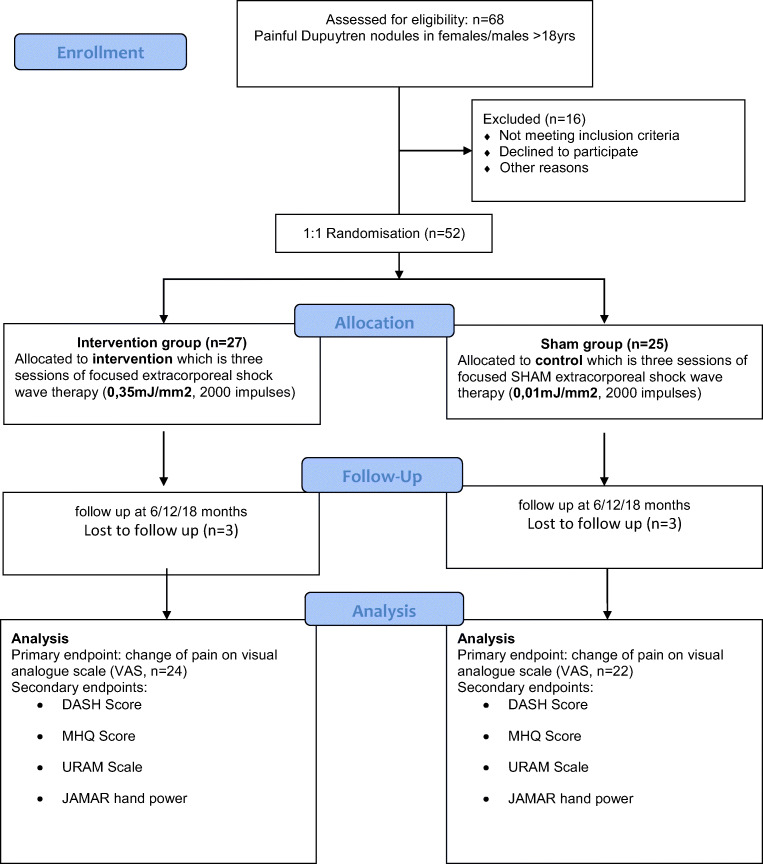
CONSORT flow chart

A total number of 52 patients with mean age of 58 ± 10 years with 62% males were randomized either to the high-energetic focused ESWT intervention group (*n* = 27) or the placebo-treatment group (*n* = 25, CONSORT flow chart Fig. 1). Detailed participants characteristics in terms of age, gender, history of Dupuytren’s or related fibromatosis, time from initial diagnosis as well as previous treatments can be found in Table 1. Mean number of Dupuytren nodules was 2.3, average time from initial diagnosis 40 months (range 3–264 months).

**Table 1:** Detailed patient’s characteristics of the Dupuyshock RCT

Variable	Intervention group (*N* = 27)	Placebo group (*N* = 25)	All patients (*N* = 52)	*P* value
Age—yr	57.6 ± 8.1	58.9 ± 10.9	58.2 ± 9.5	0.4254^+^
Male sex—no. (%)	15 (55.6)	17 (68)	35 (61.5)	0.35674*
Hand with ≥1 knot—no. (%)				0.7258
Left	20 (74.1)	18 (72)	38 (73.1)	0.86621*
Right	25 (92.6)	19 (76)	44 (84.6)	0,09754*
Both	18 (66.7)	12 (48)	30 (57.7)	0,17,342*
Number of knots	2.3 ± 1.1	2.4 ± 0.9	2.3 ± 1.0	0.9848*
Family history of Dupuytrens disease—no. (%)	9 (33.3)	8 (32)	17 (32.7)	0.91843*
Risk factors and associated conditions—no. (%)				
Exposure to vibration	0 (0)	1 (4)	1 (1.9)	0.29401*
Hand-affecting job	14 (51.9)	9 (36)	23 (44.2)	0.25017*
Hand-affecting sports	5 (18.5)			–
Hand trauma	1 (3.7)	5 (20)	6 (11.5)	0.06610*
Knuckle pads	1 (3.7)	0 (0)	1 (1.9)	0.33123*
Peyronie’s disease	2 (7.4)	0 (0)	2 (3.8)	0.16520*
Ledderhose’s disease	6 (22.2)	1 (4)	7 (13.5)	0,05441*
Diabetes	2 (7.2)	2 (8)	4 (7.7)	0.93614*
Epilepsy	0 (0)	0 (0)	0 (0)	1.0*
History of cancer	2 (7.4)	1 (4)	3 (5.8)	0.59852*
Alcohol abuse	0 (0)	1 (4)	1 (1.9)	0.29401*
Tobacco use	0 (0)	2 (8)	2 (3.8)	0.13393*
Initial diagnose—month ago				
Mean	36.4 ± 55.8	43 ± 40.3	39.6 ± 48.6	0.2444^+^
Range	3–264	4–144	3–264	–
Current medication—no. (%)				
Antihypertensive medication	6 (22.2)	4 (16)	10 (19.2)	0.56948*
Acetylcystein (ACC) treatment	3 (11.1)	2 (8)	5 (9.6)	0.70378*
Previous treatment for Dupuytren’s disease—no. (%)				
None	19 (70.4)	18 (72)	37 (71.2)	0.89689*
Surgery	3 (11.1)	5 (20)	8 (15.4)	0.37474*
Hand therapy	5 (18.5)	2 (8)	7 (13.4)	0.26686*

*Chi-quadrant test

^+^Mann-Whitney test

Patients with painful Dupuytren’s disease Tubiana stage N (nodules) or cords without flexion contractures were enrolled. The Tubiana classification—first published in 1961 [24]—distinguishes the level of total deformities by adding together the individual flexion deformity of the metacarpophalangeal (MP) joint, the proximal interphalangeal (PIP) joint, and the distal interphalangeal (DIP) joint. Six stages can be differentiated in the Tubiana classification:

- Tubiana stage 0: no lesion
- Tubiana stage N: palmar or digital nodule without established flexion deformity
- Tubiana stage 1: total flexion deformity between 0° and 45°
- Tubiana stage 2: total flexion deformity between 46° and 90°
- Tubiana stage 3: total flexion deformity between 91° and 135°
- Tubiana stage 4: total flexion deformity exceeding 135°

All patients provided written informed content. Prior to initiating treatment, the investigator identified up to three primary nodules or cords in one hand for treatment in each patient. Criteria were size of the nodule and level of pain most patients felt in this state of Dupuytren’s disease.

Inclusion criteria:

- Eligible patients are patients aged 18 or over and 80 or younger
- **Painful** Dupuytren’s disease of Tubiana stage N (nodular) and 1 involving one or more fingers or the palm only

Exclusion criteria:

- Exclusion criteria are suspected or evident pregnancy
- No painful Dupuytren’s disease
- Evident ulcerations
- No informed consent
- Age under 18 years or above 80 years

### Interventions

Patients were randomly assigned in a 1:1 ratio to either treatment with high-energetic focused electromagnetic extracorporeal shockwave therapy (focused ESWT, Storz Duolith, 2000 pulses, 0.35 mJ/m^2^ per week) or SHAM-ESWT treatment (2000 pulses, 0.01 mJ/m^2^ per week).

### Energy levels in focused extracorporeal shockwave therapy

**Fig. 2:**
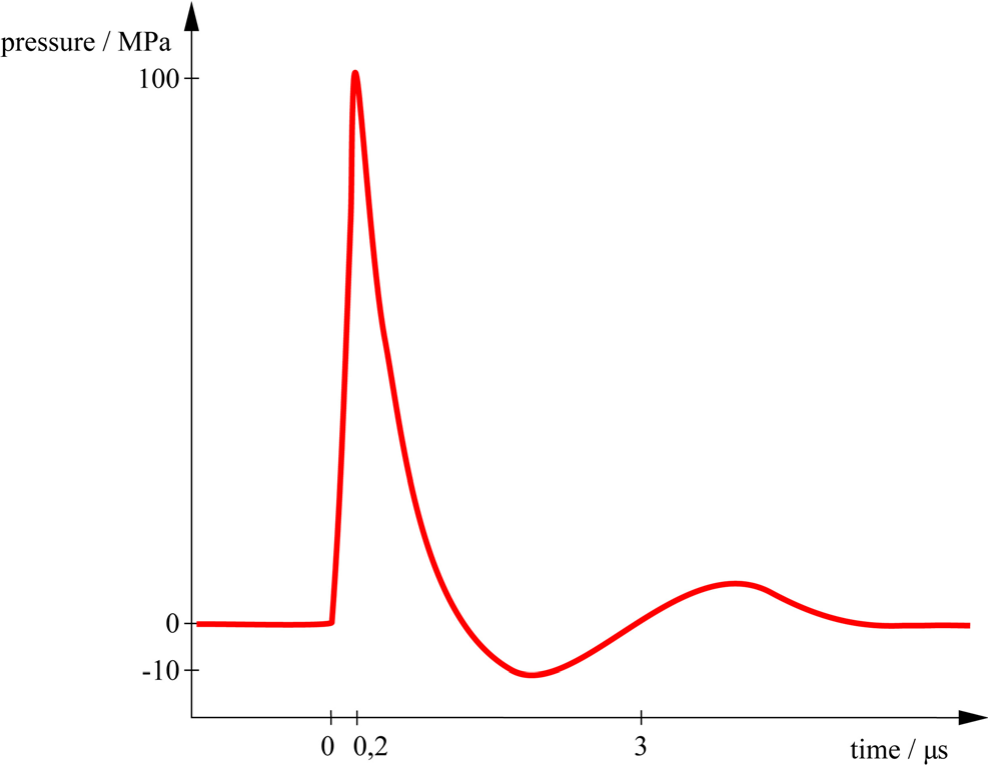
Schematic pressure profile of a focused extracorporeal shockwave (ESWT)

Extracorporeal shockwave therapy (ESWT) is using acoustic waves with a sharp, abrupt, and rapid change in pressure as a wave front with a velocity higher than the speed of sound followed by a longer negative tail to elicit a body response [25]. Generally, a shock wave can be described as a single pulse with a wide frequency range (from approx. 150 kHz up to 100 MHz), high pressure amplitude (up to 150 MPa), low tensile wave (up to −25 MPa), small pulse width and a short rise time of up to a few hundred nanoseconds (Fig. 2) (https://www.shockwavetherapy.org/about-eswt/physical-principles-of-eswt/).

On February 8, 1980, the first clinical application of extracorporeal shockwave therapy has been performed in Munich, Germany for kidney stone lithotripsy [26] [27].

From a physical point of view, focused technologies involve different generators to elicit a focused shockwave:

- Electrohydraulic generator
- Electromagnetic generator
- Piezoelectric generator

In this study, a focused electromagnetic ESWT device (Storz Duolith SD1) was used which is composed of a cylindrical coil and a parabolic reflector. A coil excites a cylindrical membrane, which generates a wave that is focused, similar to lithotripsy spark sources, by a parabolic reflector. Focused ESWT should be differentiated from radial pressures waves, which are generated either by air pressure like an air gun or by electromagnetic coils.

**Fig. 3:**
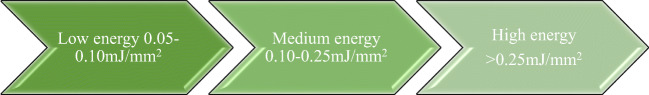
Energy flux densities (EFD measured in mJ/mm^2^) as low energetic (<0.1 mJ/mm^2^), medium energetic (0.1–0.25 mJ/mm^2^), and high energetic (>0.25 mJ/mm^2^)

By definition, energy levels of ESWT, the energy flux density (mJ/mm^2^) should be described which are defined as (Fig. 3):

- Very low/nano energetic 0.01–0.05 mJ/mm^2^
- Low energetic 0.07–0.1 mJ/mm^2^
- Medium energetic 0.1–0.25 mJ/mm^2^
- High energetic >0.25 mJ/mm^2^

The rationale for using high-energetic electromagnetic ESWT in this RCT was based on previous studies in plantar fibromatosis aka Ledderhose’s disease [16, 17], where 0.35 mJ/mm^2^ was able to reduce plantar pain significantly in a pilot cohort study. Based on these observations and pilot ESWT treatments on the palm in Dupuytren’s disease, we chose to apply high-energetic focused electromagnetic ESWT, which the patients affected from painful Dupuytren nodule tolerated without any anesthesia when the focused ESWT probe was placed directly over the nodule.

For allocation of participants, a 1:1 ratio randomization was performed using opaque envelopes for the concealment of allocation.

**Fig. 4:**
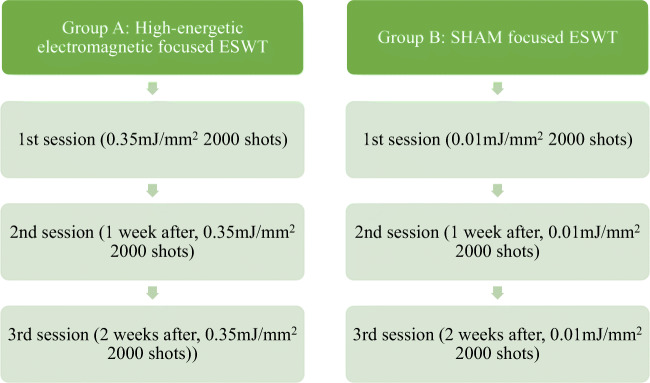
Group A with three consecutive sessions of high-energetic focused electromagnetic extracorporeal shock wave therapy (focused ESWT) vs. group B with three sessions of SHAM-ESWT on a weekly base

All patients were treated with the STORZ Duolith console with either high-energetic electromagnetic (0.35 mJ/mm^2^) or SHAM focused ESWT (0.01 mJ/mm^2^) with 2000 shots per session. A treatment cycle comprised three ESWT treatments in week 0, 1, and 2, each tied to previous examination and surveys. Follow-up examination occurred 3 months after the last treatment, continued by follow-up surveys and further questioning after 6, 12, and 18 months (Fig. 4).

### Primary and secondary end points and assessments

The primary end point of this randomized controlled trial (RCT) was a **change of pain** measured on a visual analogue scale (VAS) at follow-up *3, 6, 12, and 18 months* after the last focused shockwave treatment.

As secondary endpoints we chose validated patient-orientated outcome measures, as such as follows:

- The DASH score [28] [29]
- The Michigan Hand questionnaire (MHQ) [ 30] [31]
- The Unite Rhumatologique des Affections de la main (URAM) scale [32]

The DASH score (disabilities of the arm, shoulder and hand) consists of 30 questions on activities such as “open a tight or new jar,” “write,” “turn a key,” “prepare a meal,” and others with the patient rating on the difficulty level of such activities from 1 = no difficulty, over 2 = mild difficulty, 3 = moderate difficulty, 4 = severe difficulty, and 5 = unable to do so. The higher the score, the stronger is the deterioration of the affected upper limb. Likewise, the Michigan Hand questionnaire askes 25 unilateral and 12 bilateral questions on activities of the upper extremity. Last, the URAM scale with 9 questions was originally designed for advanced Dupuytren’s disease with contractures undergoing percutaneous needle fasciotomy. Thus, the URAM scale was evaluated in advanced Tubiana 1° or higher stages in the original description. In nodular stage like in our study, this URAM scale has not been described yet.

For language-purposed, we used the validated German translated versions of the following:

- The GERMAN-DASH [33]
- The German-MHQ [34]
- German-URAM [35] accordingly

As such, the secondary endpoints were as follows:

- An **improvement of function** measured with self-reported hand function and disability assessed by the Disabilities of Arm Shoulder and Hand Questionnaire (DASH).
- A **change** of the patient-related outcome measure Michigan Hand questionnaire (MHQ)
- A **change** of the patient-related outcome measure URAM [32]
- Hand grip strength [kg] was measured using a JAMAR dynamometer in three repetitions on each hand with elbow fully extended prior to every treatment and after the third treatment
- Patient satisfaction and progression of Dupuytren’s disease

#### Safety assessments

Monitoring of potential adverse effects was done. In case of local adverse events, patients had the opportunity to directly contact the investigator. No severe adverse events were reported during the entire study period of this RCT.

#### Statistical analysis

The statistical analysis examined the difference between the high-energetic focused ESWT and the SHAM focused ESWT (between-effect) and the repetition of measurements over the period of time (within-effect) in each patient. Metric variables were depending on the different instruments used. Mixed analysis of variation (ANOVA) had to be applied. Based on the small size of groups, standard distribution could not be taken as a basis. Accordingly, the target variable had to be transformed into a normal score via rank transformation and then converted by inverse normal transformation (INT). Afterwards, a correction of the degrees of freedom according to Greenhouse-Geiser (or Huynh-Feldt) was applied.

Eight patients of each group had to be excluded from the analysis because of missing participation in follow-up evaluation or undergoing surgery within the period of evaluation.

The statistical analysis of the pain measured with VAS shows a significant effect over the time as it shows of intervention. A significant effect over time but no effect of intervention in DASH and URAM was shown and no significant effects regarding time or intervention in MHQ.

## Results

### Primary endpoint pain in Dupuytren’s nodules

In the intervention group (3 sessions of focused electromagnetic ESWT, 0.35 mJ/mm2, 2000 shots each), pain levels were significantly reduced from 3.6 ± 1.8 to 1.9 ± 1.2 (−47%) at 3 months, to 1.4 ± 0.7 (−61%) at 6 months, to 1.7 ± 1.6 (−53%) after 12 months, and to 1.9 ± 0.8 (−47%) after 18 months in the intervention group (all *p* < 0.05).

**Fig. 5:**
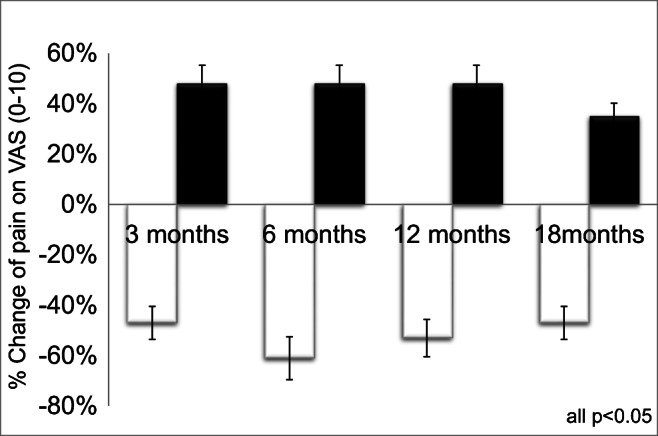
Change of pain level on visual analogue scale (VAS) after 3, 6, 12, and 18 months in the high-energetic electromagnetic focused ESWT group A (white, 0.35 mJ/mm^2^, 3 sessions) vs. SHAM-ESWT group B (black, 0.01 mJ/mm^2^, 3 sessions)

In the placebo group, pain on VAS increased from 2.2 ± 1.4 by +48% to 3.4 ± 1.7 at 3, to 3.4 ± 1.8 at 6, to 3.4 ± 1.4 at 12, and 3.1 ± 1.1 at 18 months (all *p* < 0.05, Fig. 5).

### Secondary endpoints in Dupuytren’s nodules

#### DASH score

**Fig. 6:**
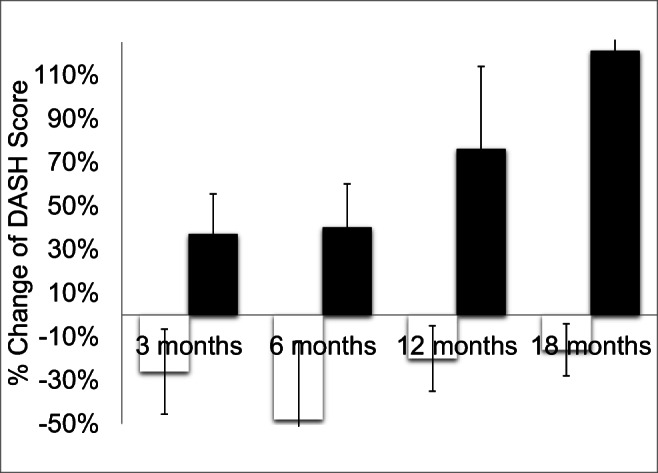
Change of DASH scores after 3, 6, 12, and 18 months in the high-energetic electromagnetic focused ESWT group A (white, 0.35 mJ/mm^2^, 3 sessions) vs. SHAM-ESWT group B (black, 0.01 mJ/mm^2^, 3 sessions)

Quality of life score tended to improve in the intervention group (DASH, 12 ± 18 to 10 ± 9) while it deteriorated in the placebo group as Dupuytren’s disease was progressing (DASH, 6 ± 10 to 14 ± 13) 18 months after treatment (Fig. 6). However, due to large standard deviation, this was not significant.

#### MHQ

**Fig. 7:**
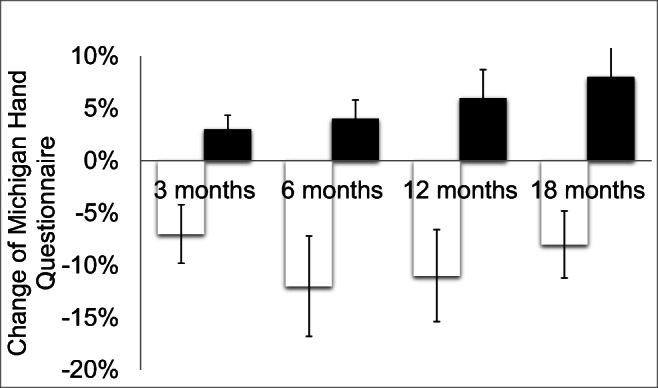
Change of MHQ scores after 3, 6, 12, and 18 months in the high-energetic electromagnetic focused ESWT group A (white, 0.35 mJ/mm^2^, 3 sessions) vs. SHAM-ESWT group B (black, 0.01 mJ/mm^2^, 3 sessions)

Michigan Hand Questionnaire (MHQ) was improved in the intervention group from 77 ± 19 to 83 ± 12, while it deteriorated in the placebo group as Dupuytren’s disease was progressing (MHQ, 79 ± 15 to 73 ± 17, Fig. 7). However, this was not significant.

#### URAM scale

Dupuytren-specific URAM scale did not change in the intervention group (3 ± 4 to 3 ± 3) while it deteriorated in the placebo group as Dupuytren’s disease was progressing (1 ± 2 to 3 ± 2). However, this was not significant.

#### Grip strength

The strength of the affected hand and fingers did not change significantly in either of the groups (Table 2).

**Table 2:** Strength testing in terms of grip strength (Jamar) and pinch strength before and after in both groups without a significant difference

Strength before and after	Intervention group (*n* = 27)	Control group (*n* = 25)
Grip strength Jamar	37 ± 12 vs. 37 ± 13 kg (n.s.)	39 ± 14 vs. 40 ± 14 kg (n.s.)
Pinch strength	9 ± 3 vs. 9 ± 3 kg (n.s.)	9 ± 3 vs. 10 ± 3 kg (n.s.)

#### Patient satisfaction

Patients’ satisfaction was higher in the intervention group for improvement of symptoms (56% vs. 12%) and reduction of disease progression (59% vs. 24%). Any Dupuytren-related intervention was performed in 26% in the intervention group and in 36% in the placebo group (n.s.) (Table 3).

**Table 3:** Patient satisfaction and progression of the Dupuytren’s disease in the intervention and the control group

	Intervention group (*n* = 27)	Control group (*n* = 25)
Improvement of symptoms	56%	12%
Any Dupuytren intervention in 18 months	25.9% (*n* = 7)	36% (*n* = 9)
Surgery	18.5% (*n* = 5)	28% (*n* = 7)
Percutaneous needle fasciotomy (PNF)	3.7% (*n* = 1)	8% (*n* = 2)
Collagenase clostridium histolyticum	3.7% (*n* = 1)	0

#### Adverse effects

No adverse events were reported.

## Discussion

The major finding of this RCT is high-energetic focused electromagnetic extracorporeal shockwave therapy (ESWT) is an effective and safe noninvasive treatment to reduce the pain in the early nodular stage of Dupuytren’s disease. Three focused ESWT sessions could demonstrate a significant pain reduction and a sustained effect over 18 months of follow-up in this randomized-controlled trial.

This finding should be discussed in detail. The nodular stage of Dupuytren’s disease is a condition which may impair activity of daily living, for example, during push-up exercises, Yoga exercises or during homework. Traditionally, the nodular stage of Dupuytren’s disease Tubiana N has not been addressed therapeutically as extensive as the Dupuytren cords at least in an evidence-based medicine point of view. Given the progressive nature of the disease, it is tempted to focus even early on in this progressive disease which might help reducing morbidity and mortality in a long-term perspective.

Nonetheless, a number of different therapeutic options have been highlighted for the nodular stage of Dupuytren’s disease; however, from an evidence-based medicine point of view, on cohort study levels mainly. Radiotherapy has been proposed since the 1980s especially in Germany for early stage of Dupuytren [36]. Randomized-controlled trials on the effect of radiotherapy in nodular Dupuytren’s disease are not published yet. A single collagenase clostridium histolyticum injection has been studied in a RCT demonstrating a significant reduction of nodule size and hardness with either 0.40 mg or 0.60 mg of collagenase [37].

### ESWT potential mechanisms

We found that three sessions of focused electromagnetic ESWT can reduce pain over a period of 18 months significantly. By now, a number of potential beneficial effects of ESWT on various tissues have been reported, such as a stem cell propagation [38], growth factor stimulation [39], anti-inflammatory actions via COX2-pathways [40], and others. Direct pain modulation via substance P or CGRP might explain part of the beneficial ESWT action in this trial [41].

**Fig. 8:**
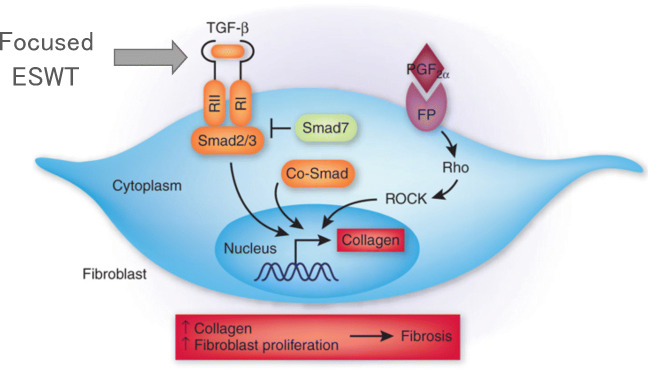
Potential antifibrotic effect of focused EWST modulating the TGF-beta receptor which drives the pro-fibrotic pathway via the Smad 2/3 pathway

A possible additional ESWT mechanism with regard to the aforementioned pain reduction in our RCT is an anti-fibrotic effect via the TGF-beta signaling pathway (Fig. 8).

TGF-ß is a master regulator of fibrosis [14]. The pro-fibrotic actions of TGF-ß are positively and negatively regulated by interactions with other signaling pathways and by noncoding RNA and epigenetic mechanisms [42]. ESWT has been shown to alter the expression of fibrosis-related molecules such as TGF-ß1, alpha smooth muscle actin (alpha-SMA), and collagen-I in human hypertropic scars [43]. Kidney function has been reported to be ameliorated by ESWT in diabetic nephropathy [44] as well as liver function in liver fibrosis [41] by ESWT. ESWT has been reported to reduce capsular fibrosis after insertion of silicone implants both, in an experimental setting [45], as well as clinically in 12 female patients undergoing breast implantation surgery [46].

Thus, it is tempting to speculate that the observed pain reduction in the ESWT intervention group with the strongest pain reduction 6 months after three focused ESWT sessions might be at least mediated by antifibrotic effects.

In addition, an anti-inflammatory effect of ESWT has been described in a number of studies [47] [48]. From a histological point of view, surgically obtained tissue samples of Dupuytren patients (mainly cords) showed a striking accumulation of immune cells with expression of leukocyte adhesion molecules as well as pro-inflammatory and pro-fibrotic cytokines [49]. Thus, focused electromagnetic high-energetic ESWT might have been acted anti-inflammatory in early stage of Dupuytren’s disease, which might support the sustained and prolonged pain reduction over 18 months in this RCT. However, both aforementioned modes of ESWT action, the antifibrotic as well as the anti-inflammatory remain speculative since this clinical RCT was not designed to elucidate the molecular level of action, since we did not obtain any tissue samples.

It is tempted to speculate that even an interaction of ESWT and nerves might play a role in this regard. Murata [50] studied the expression of activating transcription factor 3 (ATF3) and growth-associated phosphoprotein (GAP-43) as markers for nerve injury and axonal regeneration in experimental rat finding that ESWT application can lead to desensitization to the area of exposure. Hausdorf [51] found as early as in 2008 that the application of ESWT caused a statistically significant decrease in the mean number of neurons immunoreactive for substance P within the dorsal root ganglion L5 of the treated side compared with the untreated side, without affecting the total number of neurons within this dorsal root ganglion in rabbits. Therefore, focused ESWT might have exerted pain-mediated effects by direct nerve interaction via modulation of pain mediators like substance P or others.

### Limitations

While we could show a significant reduction of pain following three sessions of focused electromagnetic high-energetic ESWT, we failed to show a statistically significant improvement of the secondary outcome parameters as patient-related outcome scores. We believe this is due to the often only mild changes or impairments early stage of Dupuytren’s disease is causing reflected by our patient cohort with very low scoring in the patient-related outcome measures DASH, MHQ, and URAM. All questionnaires included (DASH, MHQ, URAM) revealed at baseline only mild changes from normal in our patient cohort (DASH mean 12 points (0–100); MHQ 78 points (0–100)); therefore, the potential benefit of a given intervention (here focused high-energetic ESWT) might be hampered by the only mild nature of impairment. For the DASH, the minimal clinically important difference has been reported to be 11 points in an Italian study and 15 points by the DASH authors [52]. We observed 12 points at baseline and 6 points at 6-months follow-up in the intervention group.

For the Michigan Hand Questionnaire (MHQ), this so called “ceiling effect” has been reported by the founder of the MHQ, Kevin Chung [53] with points > 75 in the MHQ—initial high scores (> 75points in MHQ) prevented the post-surgical scores from showing high improvement. We determined 77 ± 19 points before and 87 ± 10 points at 6-months follow-up in the intervention group.

## Conclusion

Focused electromagnetic high-energetic ESWT can significantly reduce pain in painful nodules in Dupuytren’s disease. No adverse effects were noted.
